# Specificity Protein 2 (Sp2) Is Essential for Mouse Development and Autonomous Proliferation of Mouse Embryonic Fibroblasts

**DOI:** 10.1371/journal.pone.0009587

**Published:** 2010-03-08

**Authors:** Frank Baur, Kerstin Nau, Dennis Sadic, Lena Allweiss, Hans-Peter Elsässer, Nynke Gillemans, Ton de Wit, Imme Krüger, Marion Vollmer, Sjaak Philipsen, Guntram Suske

**Affiliations:** 1 Institut für Molekularbiologie und Tumorforschung, Philipps-Universität Marburg, Marburg, Germany; 2 Institut für Zytobiologie und Zytopathologie, Philipps-Universität Marburg, Marburg, Germany; 3 Department of Cell Biology, Erasmus MC, Rotterdam, The Netherlands; University of Birmingham, United Kingdom

## Abstract

**Background:**

The zinc finger protein Sp2 (specificity protein 2) is a member of the glutamine-rich Sp family of transcription factors. Despite its close similarity to Sp1, Sp3 and Sp4, Sp2 does not bind to DNA or activate transcription when expressed in mammalian cell lines. The expression pattern and the biological relevance of Sp2 in the mouse are unknown.

**Methodology/Principal Findings:**

Whole-mount in situ hybridization of mouse embryos between E7.5 and E9.5 revealed abundant expression in most embryonic and extra-embryonic tissues. In order to unravel the biological relevance of Sp2, we have targeted the *Sp2* gene by a tri-*loxP* strategy. Constitutive *Sp2null* and conditional *Sp2cko* knockout alleles were obtained by crossings with appropriate Cre recombinase expressing mice. Constitutive disruption of the mouse Sp2 gene (*Sp2null*) resulted in severe growth retardation and lethality before E9.5. Mouse embryonic fibroblasts (MEFs) derived from *Sp2null* embryos at E9.5 failed to grow. Cre-mediated ablation of Sp2 in *Sp2cko/cko* MEFs obtained from E13.5 strongly impaired cell proliferation.

**Conclusions/Significance:**

Our results demonstrate that Sp2 is essential for early mouse development and autonomous proliferation of MEFs in culture. Comparison of the Sp2 knockout phenotype with the phenotypes of Sp1, Sp3 and Sp4 knockout strains shows that, despite their structural similarity and evolutionary relationship, all four glutamine-rich members of the Sp family of transcription factors have distinct non-redundant functions in vivo.

## Introduction

Specificity proteins (Sps) are transcription factors that control the expression of a variety of different genes including house keeping, tissue-specific, development-specific and cell-cycle-regulated genes (reviewed in [1]–[4]). Nine different Sp proteins, designated Sp1 to Sp9, have been identified in mammals [4]. They share a highly conserved zinc finger DNA-binding domain comprising three zinc fingers at the C-terminus (ZNF), the adjacent Buttonhead-box (Btd-box: CXCPXC), and the N-terminal Sp-box, a stretch of conserved amino acids (SPLALLAATCSK/RIG/E) of unknown function. The Sp subclass members Sp1, Sp2, Sp3 and Sp4 are further characterized by N-terminal glutamine-rich domains, whereas Sp5 to Sp9 contain proline, alanine or serine/threonine-rich domains [1], [4]. Sp1 and Sp3 are ubiquitously expressed, whereas Sp4 is most prominently found in neuronal tissues [1]–[3]. Sp1, Sp3 and Sp4 recognize the same promoter elements (GC- and GT-boxes) with similar specificity and affinity [5].

Gene targeting experiments revealed that individual Sp family members differ in their biological function, as they exhibit distinct phenotypes. S*p1null* embryos die around embryonic day (E) 10.5 [6]. *Sp3null* mice develop until the end of pregnancy but die immediately after birth due to respiratory failure [7]. *Sp3null* embryos suffer from a variety of defects including impaired skeletal bone ossification, tooth development [7], [8], cardiac development [9], and placenta organisation [10]. Newborn Sp4-deficient mice do not show obvious abnormalities [11], [12]. However, two-thirds of the mice die within the first month after birth for unknown reasons. Surviving Sp4-deficient animals are growth-retarded [11], [12]. Furthermore, both the male and female Sp4 *null* mice have problems in reproduction; males do not breed and females are delayed in sexual maturation [11], [12]. Finally, Sp4 is required for specification of the cardiac conduction system [13], [14].

Sp2 is the least characterized member of the glutamine-rich subgroup of Sp factors. Originally, Sp2 was cloned by virtue of the similarity of its zinc finger domain with the zinc finger region of Sp1 [15]. Despite this similarity, Sp2 overexpressed in insect or mammalian cells binds poorly to DNA, and has little or no capacity to stimulate transcription from promoters that are activated by other Sp family members [16]. Consistently, endogenous Sp2 DNA-binding activity in nuclear extracts prepared from cells that express abundant amounts of Sp2 was not observed ([16], and our own unpublished results). Sp2 is associated with the nuclear matrix and localizes predominantly within subnuclear foci that are distinct from promyelocytic oncogenic domains [17]. However, the functional significance of this observation remains unclear.

Towards understanding the physiological relevance of Sp2, we targeted the *Sp2* gene and generated Sp2 loss-of-function mutants. *Sp2null* embryos survive until E9.5 of gestation. They are severely retarded in growth and show a broad range of phenotypic abnormalities. Mouse embryonic fibroblasts (MEFs) derived from *Sp2null* embryos failed to grow indicating a cell-autonomous role of Sp2 in the control of cellular proliferation. This suggestion was corroborated by MEFs obtained from embryos carrying floxed Sp2 alleles (*Sp2cko/cko*). Cre-mediated ablation of Sp2 in *Sp2cko/cko* MEFs resulted in a strong decrease of proliferation. Collectively, we conclude that Sp2 is required for early mouse development and autonomous proliferation of MEFs in culture.

## Results

### Expression of *Sp2* in the Mouse Embryo

**Figure 1:**
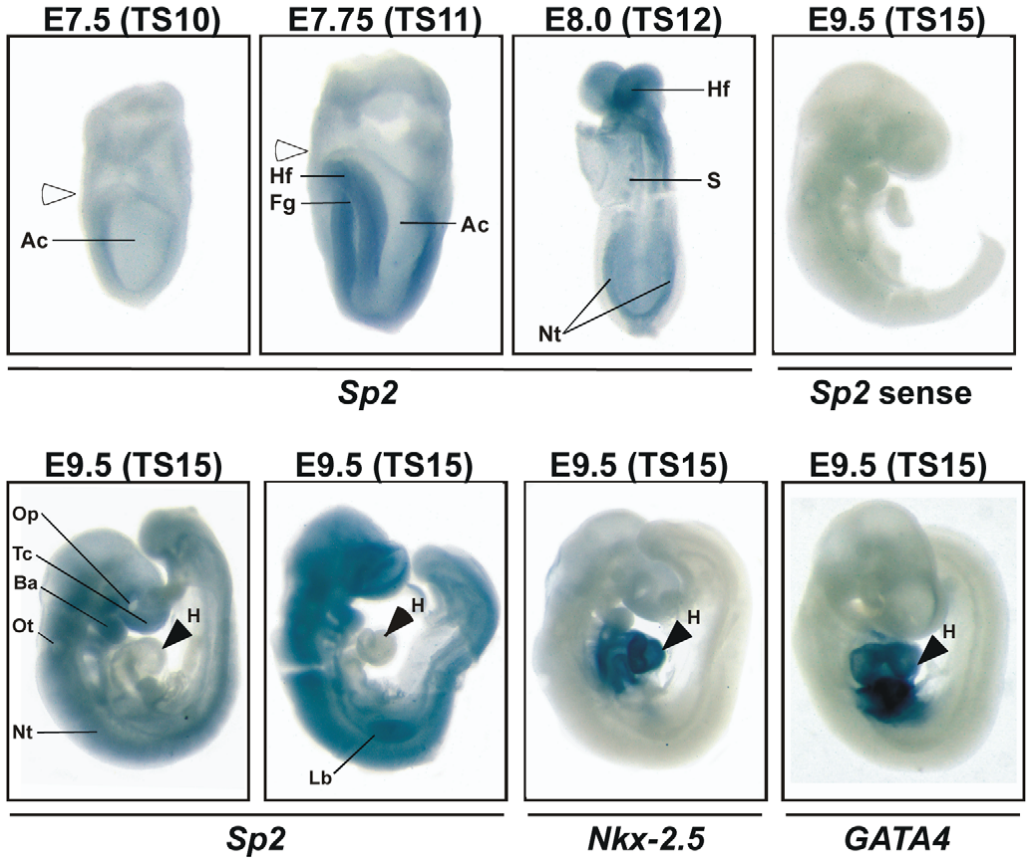
*Sp2* mRNA expression in mouse embryos. Whole-mount *in situ* hybridization on embryos at the indicated developmental Theiler stages (E7.5-TS10, E7.75-TS11, E8.0-TS12 and E9.5-TS15) with the indicated probes (*Sp2*, *Sp2* sense, *Nkx-2.5* and *GATA4*). The two embryos at E9.5 (TS15) represent two independent *in situ* hybridization experiments with embryos obtained from different litters. Ac, amniotic cavity; Ba, first branchial arch; Hf, headfold; Fg, foregut; H, heart; Lb, limb bud; Nt, neuronal tissue; Op, optic pit; Ot, otic vesicle; S, somites; Tc, telencephalon. The white arrowheads denote the boundary between embryonic and extraembryonic tissue.

We analyzed expression of the zinc-finger transcription factor *Sp2* at early embryonic stages by whole-mount *in situ* hybridization on E7.5 to E9.5 mouse embryos (Figure 1). At Theiler stage TS10 (E7.5) Sp2 is similarly expressed in embryonic as well as in extra-embryonic tissues. At TS11 and TS12 Sp2 expression is markedly increased in the embryonic tissues especially in the headfold. At a later stage (TS15) strong expression of Sp2 is observed throughout the embryo with exception of the heart. Control hybridizations with the heart-specific markers *Nkx2-5* and *GATA4* confirmed the accessibility of the heart for hybridization probes (Figure 1). Strongest expression at this stage occurs in the neural tissues, the neuroepithelium surrounding the optical and otic vesicles, the first branchial arch and the forelimb buds and the auditory pit (Figure 1). Taken together, our in situ hybridization experiments indicate abundant expression of Sp2 in most embryonic and extra-embryonic tissues during development.

### Targeting of the Mouse *Sp2* Gene

**Figure 2:**
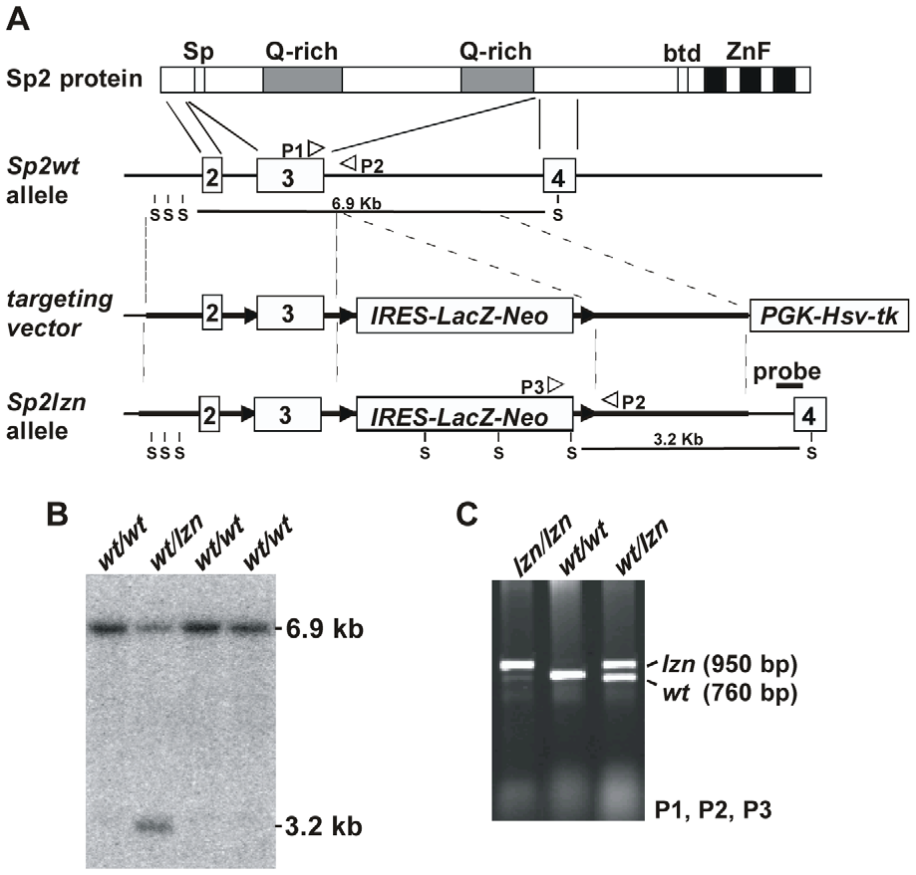
Targeting strategy for the mouse *Sp2* gene. A) Schematic representation of the Sp2 protein structure, the wild type *Sp2* allele, the targeting vector and the targeted *Sp2lzn* allele. The Sp-box (Sp), two glutamine-rich domains (Q-rich), the btd-box (btd) and the three zinc fingers (ZnF) of the Sp2 protein are indicated. Connecting lines with the corresponding murine *Sp2* gene region indicate the derivation of N-terminal parts of the Sp2 protein. The Sp-box and two glutamine-rich regions are encoded by exon 3. In the targeted *Sp2* locus exon 3 and the *IRES-lacZ-neo* cassette are floxed (black arrowheads). White arrowheads depict PCR primer locations (P1, P2 and P3). S, SacI sites. B) Southern blot analysis of transfected ES cells for the *Sp2wt* allele (6.9 kb) and the targeted *Sp2lzn* allele (3.2 kb) using SacI-restricted genomic DNA and the 387 bp probe indicated in Figure 2A. C) PCR analysis of E13.5 embryos for the *Sp2lzn* allele with the indicated primer combination (P1, P2 and P3).

To investigate the role of Sp2 in development we generated Sp2 knockout mice by homologous recombination in embryonic stem cells. The open reading frame of the *Sp2* gene encodes a polypeptide of 612 amino acids with a predicted molecular weight of 65 kDa. The entire amino acid sequence of Sp2 is encoded by seven exons. Concerned that the loss of Sp2 might produce early lethality, we employed a tri-*loxP* strategy to generate *Sp2null* mice and *Sp2* floxed mice, which could be used for conditional knockout experiments. Similar to strategies that we used previously for targeting the *Sp3* and *Sp4* genes, we targeted exon 3, a large exon that encodes for most of the N-terminal part of Sp2 including the Sp-box and two glutamine-rich regions (Figure 2A). As *Sp2* mRNA is expressed in mouse ES cells (data not shown) the targeting strategy allowed expression of a *lacZ-neo* fusion gene under the control of the endogenous *Sp2* promoter (Figure 2A). Southern blot (Figure 2B) and PCR analyses (Figure 2C) revealed successful homologous recombination in 8 out of 80 ES cell clones analyzed.

**Figure 3:**
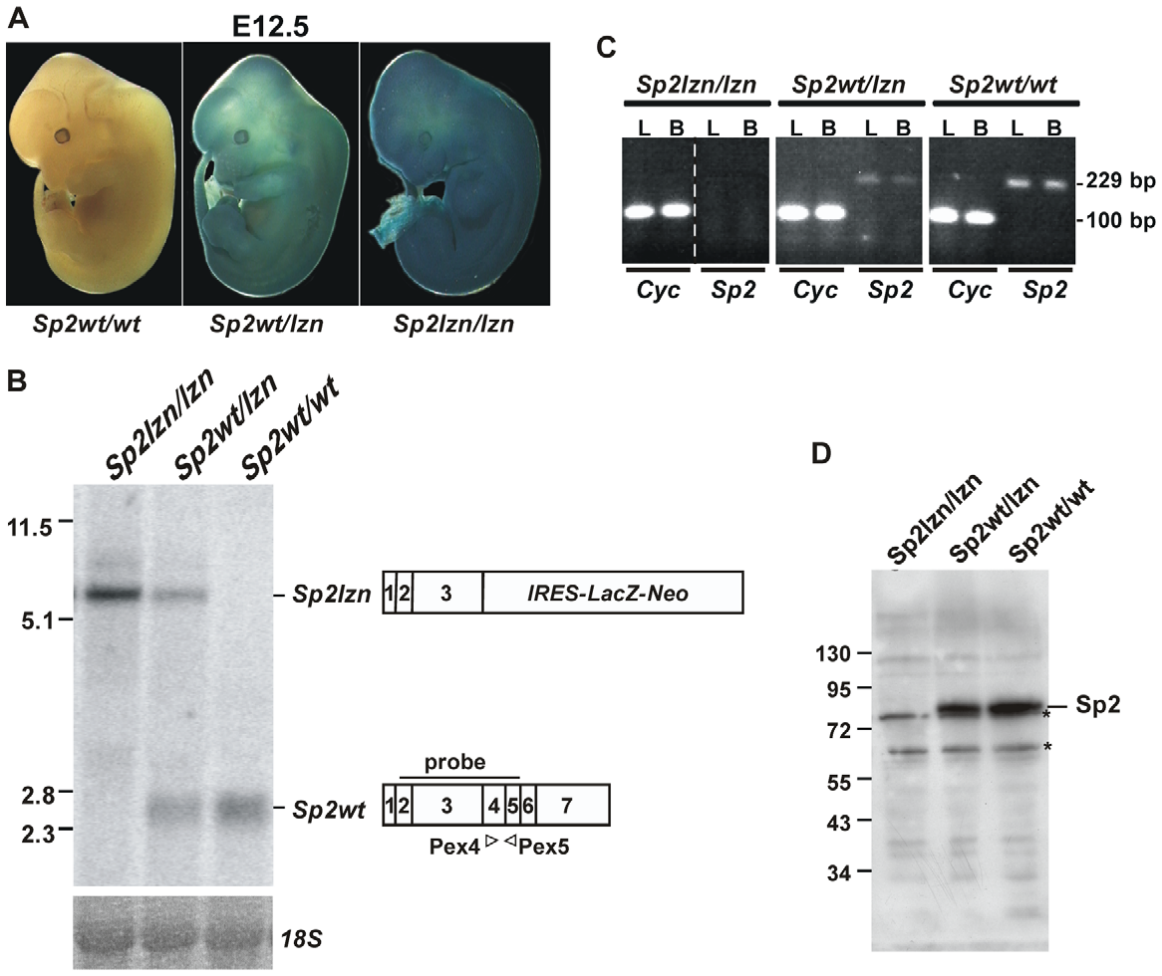
Aberrant Sp2 mRNA expression in *Sp2lzn/lzn* mice. A) Whole-mount lacZ staining of E12.5 embryos with the indicated genotypes. B) Northern blot analysis of total RNA extracted from E18.5 embryos. RNA size markers are indicated on the left side. The *18S* rRNA signal depicts the loading control. The wild type *Sp2* mRNA and the aberrant *Sp2-IRES-LacZ-Neo* mRNA are drawn schematically along with the Northern probe and the primers Pex4 and Pex5 used for the PCR analysis shown in Figure 3C. The numbers 1 to 7 indicate the derivation of mRNA sequences from exons. C) Absence of RNA derived from exons 4 and 5 in *Sp2lzn/lzn* mice. RT-PCR was performed with RNA from liver (L) and brain (B) of *Sp2lzn/lzn*, *Sp2wt/lzn* and *Sp2wt/wt* embryos using the primers Pex4 and Pex5 indicated in Figure 3B. Amplification of cyclophilin (*Cyc*) mRNA sequences was used as a positive control. D) Western blot analysis of Sp2 using extracts from primary mouse embryonic fibroblasts derived from E13.5 embryos with the indicated genotypes. The asterisks denote cross-reacting proteins that serve as internal loading control.

Mice heterozygous for the targeted *Sp2* allele (*Sp2wt/lzn*) were obtained after injection of ES cells into blastocysts and subsequent crossing of the chimeric animals with wild type mice. Sp2*wt/lzn* mice are viable, reproduce normally and display no obvious abnormalities. Whole-mount LacZ staining on E12.5 embryos confirmed the notion that Sp2 is widely if not ubiquitously expressed during development (Figure 3A). As we expected that the insertion of the *IRES-lacZ-neo* cassette would result in impaired Sp2 expression, we crossed *Sp2wt/lzn* mice to homozygosity. Northern blot analyses revealed aberrant *Sp2* mRNA expression (Figure 3B). The 7 kb transcript expressed from the *Sp2lzn* allele represents an RNA species containing exon 1 to 3 of the *Sp2* gene fused to *LacZ-neo* sequences. RT-PCR analysis revealed that regions downstream of exon 3 are not expressed from the *Sp2lzn* allele (Figure 3C). The presence of an mRNA species containing *Sp2* coding sequences was expected to result in expression of an N-terminal Sp2 fragment. Immunoblot analysis revealed the absence of the full-length Sp2 protein; a truncated Sp2 N-terminal fragment, however, was not detected (Figure 3D). Control experiments with recombinant Sp2 fragments showed that our Sp2 antiserum can detect the N-terminal part of Sp2 (data not shown). Likely, expression of the Sp2 N-terminal domain in *Sp2lzn/lzn* mice is below the detection limit.

### Impaired Development of Targeted *Sp2lzn/lzn* Mice

**Figure 4:**
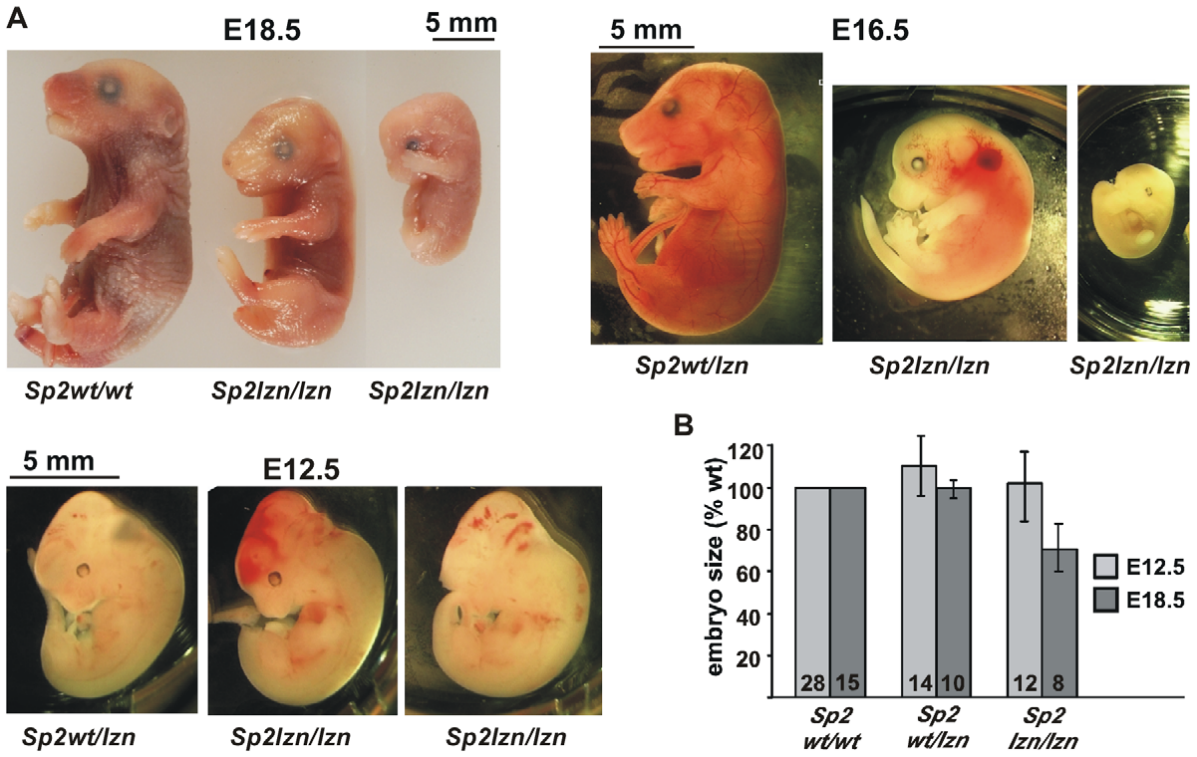
Impaired development of *Sp2lzn/lzn* embryos. A) *Sp2lzn/lzn* embryos at E18.5, E16.5 and E12.5 representing typical phenotypic variants. *Sp2lzn/lzn* embryos at E12.5 have a normal body size but display hemorrhaging predominantly at the head. B) Embryo size distribution at E12.5 and E18.5. The number of embryos analyzed is indicated at the bottom of the bars.

Genotyping of 91 10-day-old pups from heterozygous *Sp2wt/lzn* intercrosses revealed wild type and heterozygous animals at the expected 1∶2 ratio. Only one homozygous *Sp2lzn/lzn* mutant pup was found. This pub died before genotyping was finished and could not be examined. We then examined intercross embryos at E9.5 through E18.5 (Table 1). Overall, *Sp2lzn/lzn* embryos at E16.5 and E18.5 were smaller than their wild type and heterozygous littermates. They were generally developmentally retarded, albeit with considerable variation (Figure 4). Moreover, many of the *Sp2lzn/lzn* embryos were already dead at these developmental stages. In contrast, Sp2*lzn/lzn* embryos at E12.5 had a normal body size. However, they displayed hemorrhaging, predominantly in the head area (Figure 4). At E9.5 *Sp2lzn/lzn* embryos appeared apparently normal. Taken together the various developmental defects displayed by *Sp2lzn/lzn* mutant embryos indicate that Sp2 is required for normal embryonic development at late embryonic stages.

**Table 1:** Genotype distribution of *Sp2wt/lzn* intercrossings.

*Sp2wt/lzn x Sp2wt/lzn*			*Sp2wt/wt*	*Sp2wt/lzn*	*Sp2lzn/lzn*
Born	n = 91	(100%)	26 (28.6%)	64 (70.3%)	1* (1.1%)
E18.5	n = 54	(100%)	13 (24.1%)	31 (57.4%)	10 (18.5%)
E16.5	n = 13	(100%)	3 (23.1)	7 (53.8%)	3 (23.1)
E13.5	n = 72	(100%)	21 (29.2%)	36 (50.0%)	15 (20.8%)
E12.5	n = 106	(100%)	27 (25.5%)	50 (47.2%)	29 (27.4%)
E9.5	n = 51	(100%)	16 (31.4%)	22 (43.1%)	13 (25.5%)

P, postnatal day; E, day of embryonic development; n, number of mice/embryos.

*died two weeks after birth.

### Early Embryonic Lethality of *Sp2null* Mice

**Figure 5:**
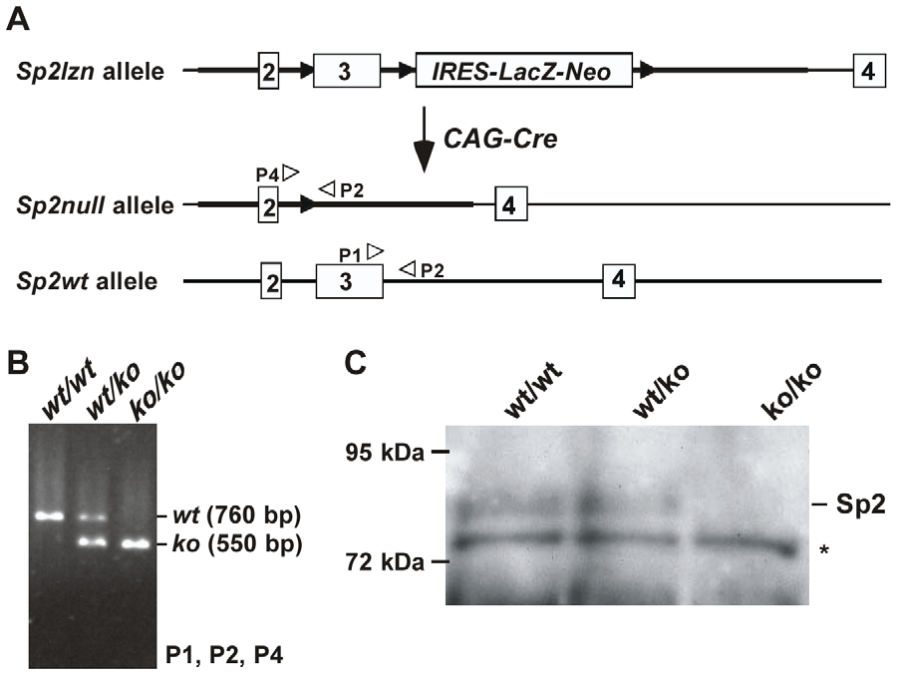
Generation of *Sp2null* mice. (A) Mice carrying an *Sp2null* allele were obtained by crossing heterozygous *Sp2wt/lzn* mice with *CAG-Cre* mice [18] thereby removing the selection marker (*IRES-LacZ-Neo*) and exon 3. Black arrowheads depict *loxP* sites, white arrowheads indicate PCR primer locations. B) PCR analysis of the *Sp2* wild type (*wt*) and the knockout (*ko*) allele using primers P1, P2 and P4 indicated in panel A. C) Immunoblot analysis of Sp2 in extracts of primary mouse embryonic fibroblasts derived from E9.5 embryos with the indicated genotype. The asterisk denotes a cross-reacting protein.

Northern blot and PCR analyses of tissues form *Sp2lzn/lzn* embryos revealed aberrant mRNA expression from the targeted *Sp2* locus. As expression of small amounts of N-terminal sequences of Sp2 could result in a hypomorphic phenotype we generated *Sp2null* mice by crossing heterozygous Sp2*wt/lzn* animals with *CAG-Cre* mice that express the Cre recombinase under control of the cytomegalovirus immediate early enhancer-chicken β-actin hybrid (CAG) promoter [18]. The resulting heterozygous *Sp2wt/ko* mice were healthy and fertile, and were intercrossed to generate *Sp2null* mice (Figure 5). Surprisingly, the phenotype of *Sp2null* embryos differs markedly from that of Sp2*lzn/lzn* embryos, strongly suggesting that the insertion of the *LacZ-neo* fusion gene into the third intron did not disrupt Sp2 functions completely.

No Sp2*null* embryos were found alive beyond E10.5 (Table 2). The few *Sp2null* embryos recovered at E12.5 to E13.5 were almost completely reabsorbed. Embryos at E9.5 and earlier stages displayed genotype distributions close to the expected Mendelian ratios (Table 2).

**Table 2:** Genotype distribution of *Sp2wt/ko* intercrossings.

*Sp2wt/ko x Sp2wt/ko*			*Sp2wt/wt*	*Sp2wt/ko*	*Sp2null*
P10	n = 81	(100%)	34 (42%)	47 (58%)	0 (0%)
E17.5	n = 7	(100%)	3 (42.9%)	4 (57.1%)	0 (0%)
E13.5	n = 8	(100%)	0 (0.0%)	8 (88.9%)	1* (11.1%)
E12.5	n = 23	(100%)	7 (26.9%)	16 (61.5%)	3* (11.5%)
E10.5	n = 39	(100%)	18 (46.2%)	20 (51.3%)	1 (2.6%)
E9.5	n = 303	(100%)	69 (22.8%)	166 (54.8%)	68+ (22.4%)
E8.5	n = 34	(100%)	10 (29.4%)	16 (47.1%)	7 (20.6%)
E7.5	n = 9	(100%)	1 (11.1%)	6 (66.7%)	2 (22.2%)

P, postnatal day; E, day of embryonic development; n, number of mice/embryos.

*embryos were dead and reabsorbed.

+ 7 out of 68 embryos were reabsorbed.

**Figure 6:**
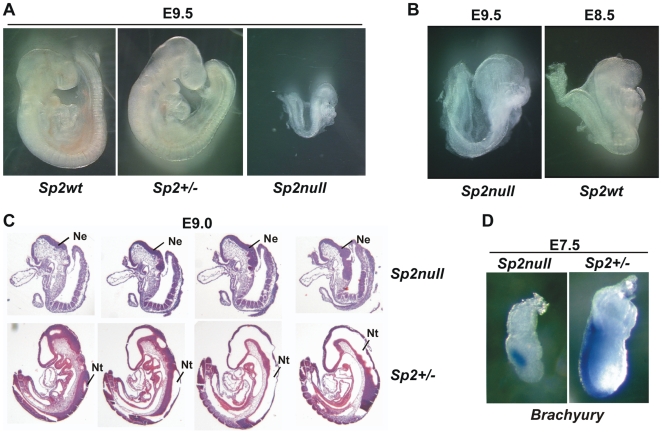
Phenotype of *Sp2null* embryos. A) An *Sp2null* embryo at E9.5 in comparison to wild type (*Sp2wt*) and heterozygous (*Sp2+/−*) littermates. B) An *Sp2null* embryo at E9.5 in direct comparison to an E8.5 wild type embryo (*Sp2wt*). C) Serial longitudinal sections of heterozygous *Sp2* (*Sp2+/−*) and *Sp2null* embryos from day E9.0. *Sp2null* embryos are smaller, have not turned and have an open cranial neural tube (Nt). Ne, neural epithelium. D) Whole-mount in situ hybridization on E7.5 embryos using the mesodermal marker *Brachyury* as a probe.

*Sp2null* embryos developed until the nine somite stage but failed to undergo body turning. At E9.5 *Sp2null* embryos were much smaller than their littermates (Figure 6A). However, heartbeat and superficial attachment of the allantois to the chorion were observed. Generally, the appearance of E9.5 *Sp2null* embryos was very similar to E8.5 wild type and heterozygous embryos (Figure 6B) indicating a general developmental delay. This conclusion is supported by serial longitudinal sections that revealed that E9 Sp2 knockout embryos have an open cranial neural tube (Figure 6C) typical for E8 wild type or *Sp2* heterozygous embryos.

Growth and developmental retardation of Sp2-deficient embryos were already visible at E7.5 exemplified by the expression pattern of the mesodermal marker *Brachyury*. At this stage wild type and heterozygous Sp2 embryos displayed intense *Brachyury* expression. Expression of *Brachyury* in the smaller *Sp2null* littermates, however, was very weak (Figure 6D) resembling expression of *Brachyury* in the nascent primitive streak at the onset of gastrulation of E6.5 embryos [19], [20].

### Generation of Conditional Sp2 Knockout Mice (*Sp2cko/cko* Mice)

**Figure 7:**
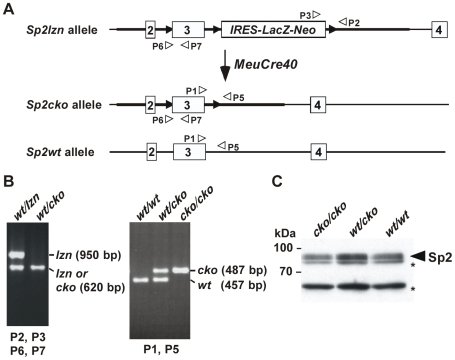
Generation of conditional *Sp2* mice (*Sp2cko/cko* mice). A) Crossing of heterozygous *Sp2wt/lzn* mice with *MeuCre40* mice resulted in generation of an *Sp2cko* allele in which the *lacZ-Neo* fusion gene is absent. B) PCR analysis of the *Sp2* wild type (*wt*), *Sp2-IRES-lacZ-Neo* (*lzn*) and *Sp2cko* (*cko*) alleles with the indicated primers. C) Western blot analysis of Sp2 in extracts of *Sp2cko/cko*, *Sp2cko/wt* and *Sp2wt/wt* MEFs.

As inactivation of Sp2 is mid-gestational lethal we decided to generate mice with floxed exon 3 lacking the *IRES-LacZ-neo* cassette that would allow inactivating Sp2 in a stage-specific manner as well as in cell lines derived from these mice. Generation of such a conditional Sp2 knockout allele (*Sp2cko* allele) could be accomplished by selective removal of the *IRES-lacZ-neo* cassette upon partial Cre-*loxP* recombination (Figure 7). Injection of fertilized eggs with different concentrations of *Cre* RNA [21] resulted either in complete excision of exon 3 and the *IRES-lacZ-neo* cassette or excision of exon 3, but never in excision of only the *IRES-lacZ-neo* cassette. Therefore, we employed an *in vivo* approach.

*MeuCre40* (*m*osaic-*e*arly embryonic-*u*biquitous *Cre* transgene), a transgenic mouse line that has been shown to generate partial mosaic Cre-*loxP* recombination patterns in the early embryo [22], were crossed with heterozygous *Sp2wt/lzn* mice. This resulted in mosaicism for recombination events at the *Sp2lzn* locus that was transmitted through the germline. After back crossing mosaic animals to wild type mice we found a single female, out of 130 progeny analysed, that carried an allele with the desired floxed exon 3 but lacking the *IRES-lacZ-neo* cassette (*Sp2wt/cko*) (Figure 7B), the *Sp2null* allele and *Cre* recombinase. Crossing of the single *Sp2wt/cko* female with wild type mice resulted also in male *Sp2wt/cko* animals.

Homozygous *Sp2cko/cko* mice in which both *Sp2* alleles are floxed, were obtained by intercrossing of *Sp2wt/cko* mice. Consistent with wild type Sp2 protein expression from the floxed *Sp2* locus (Figure 7C), *Sp2cko/cko* mice are phenotypically indistinguishable from wild type animals.

### Sp2 Is Essential for Proliferation of Mouse Embryonic Fibroblasts

**Figure 8:**
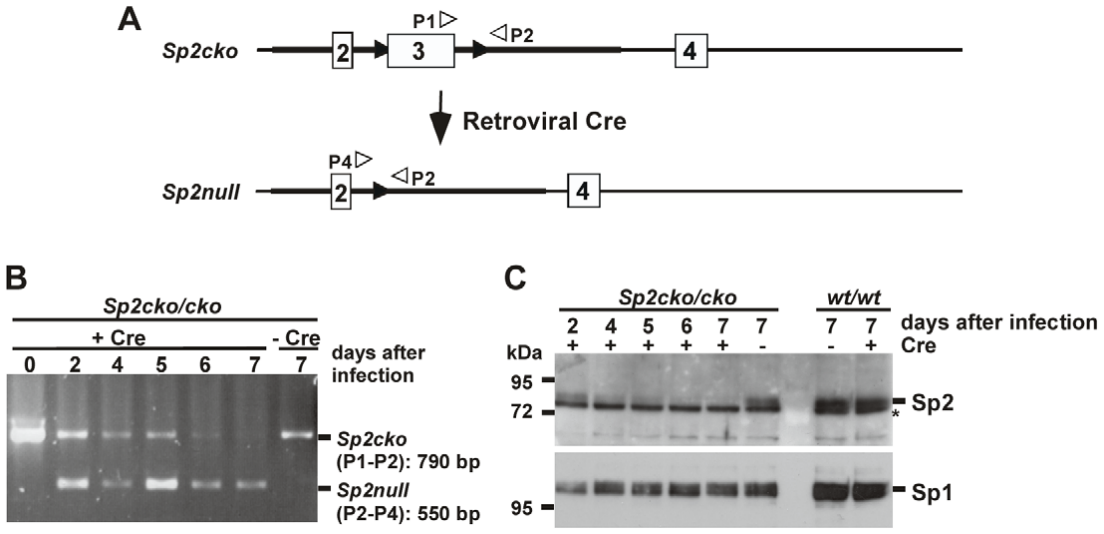
Depletion of Sp2 in *Sp2cko/cko* MEFs upon retroviral Cre-infection. A) Schematic presentation of the *Sp2cko* allele and the *Sp2null* allele obtained after retroviral Cre infection along with the positions of the primers used for genotyping. B) PCR analysis of Cre-infected *Sp2cko/cko* MEFs at various time points post-infection as indicated. C) Western blot analysis of Sp2 in Cre-infected *Sp2cko/cko* and *Sp2wt/wt* MEFs at various time points post-infection as indicated. The asterisk indicates a cross-reacting protein. To control for loading, the same blot was re-stained for Sp1.

Mouse embryonic fibroblasts (MEFs) obtained from E9.5 *Sp2null* embryos became flat-shaped and did not divide, whereas wild type and heterozygous MEFs derived from siblings grew normally. The lack of proliferation of MEFs obtained from E9.5 *Sp2null* embryos could reflect the developmental retardation of these embryos or could be due to the lack of Sp2. To explore directly the potential role of Sp2 in cellular proliferation of MEFs we isolated MEFs carrying floxed *Sp2* alleles (*Sp2cko/cko*) from E13.5 embryos. *Sp2cko/cko* MEFs and corresponding wild type and heterozygous MEFs (*Sp2wt/cko*) were immortalized by serial passages of primary MEFs until they pass their growth-crisis stage. Infection of *Sp2cko/cko* MEFs with a retrovirus expressing Cre recombinase resulted in a time-dependent deletion of the floxed *Sp2* alleles and complete loss of the Sp2 protein at seven days post-infection (Figure 8).

**Figure 9:**
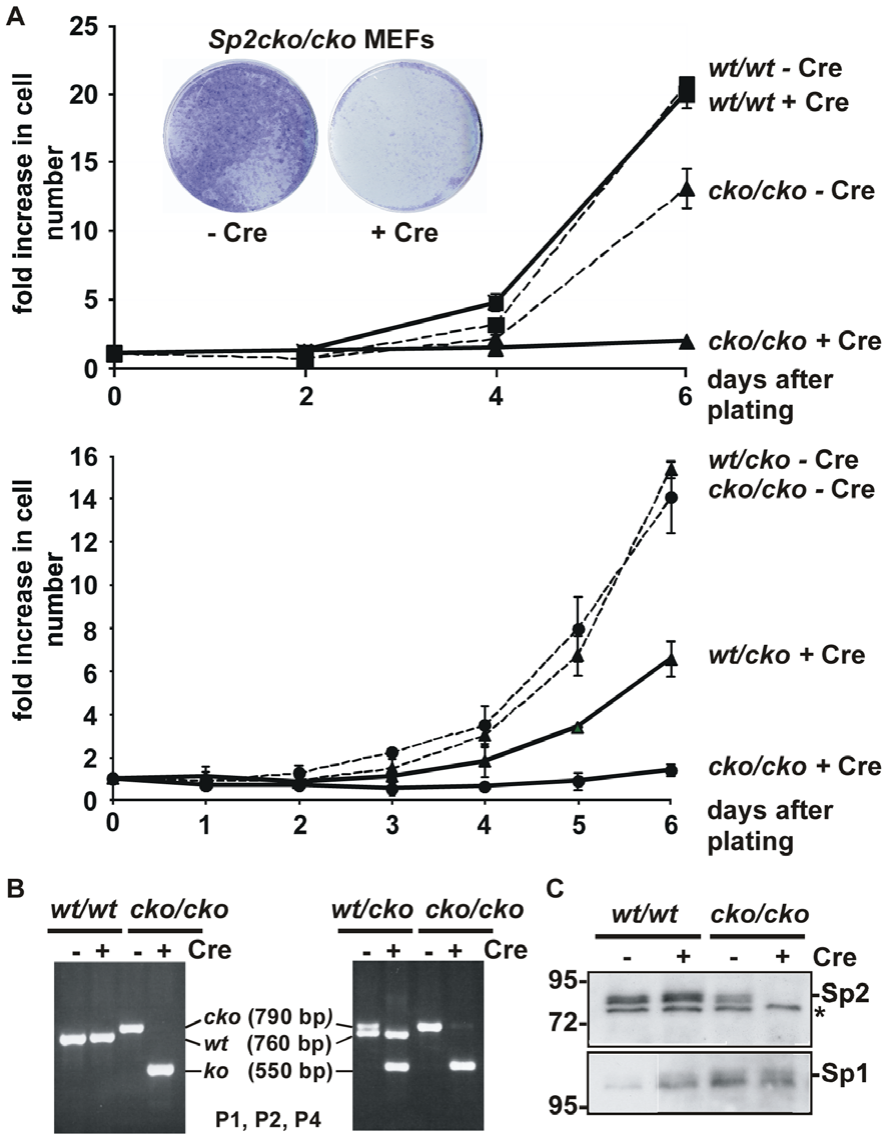
Sp2 is required for proliferation of MEFs. A) MEFs with the indicated genotypes were infected with a Cre-expressing (+ Cre) or an empty control (- Cre) retrovirus, selected with puromycin for seven days and subsequently replated for determination of growth curves. The two growth curves were performed with two different *Sp2cko/cko* lines. Results are presented as fold increase in cell number relative to time 0. The insert in the upper panel shows a crystal violet staining of *Sp2cko/cko* MEFs infected with an empty retrovirus (- Cre) or a Cre-expressing retrovirus (+ Cre). B) PCR analyses of genomic DNA from *Sp2wt/wt*, *Sp2wt/cko* and *Sp2cko/cko* MEFs at day 0 of the growth curve determinations using the primer combination P1, P2, P4. C) Immunoblot analysis of Sp2 with MEF extracts of the indicated genotype.

Consistent with previous reports [23], [24] continuous Cre expression in wild type cells slightly decreased growth (Figure 9). However, Cre-mediated ablation of Sp2 in *Sp2cko/cko* MEFs resulted in strongly reduced proliferation relative to control retrovirus-infected cells (Figure 9). This finding together with the observation that MEFs derived from *Sp2null* embryos do not proliferate suggest a role of Sp2 in the control of cellular proliferation.

**Figure 10:**
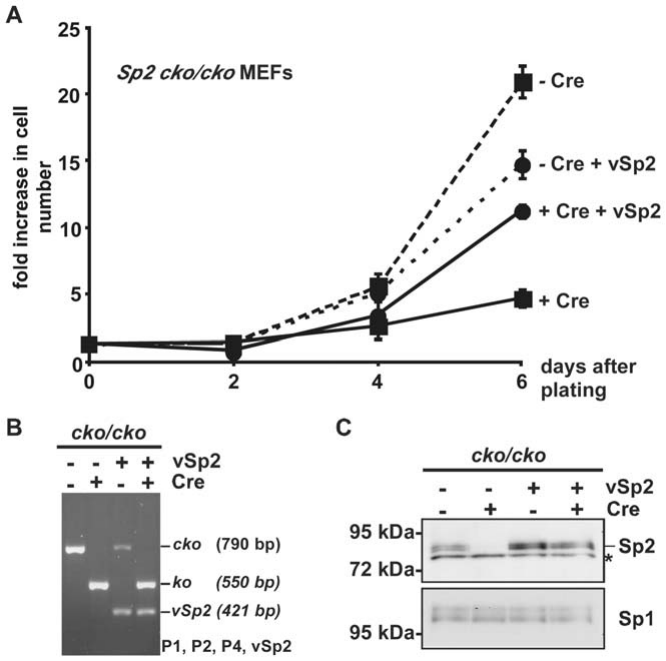
Partial rescue of proliferation by expression of exogenous Sp2. A) *Sp2cko/cko* MEFs expressing exogenous Sp2 (vSp2) were infected with an empty control (- Cre) or a Cre-expressing retrovirus (+ Cre), selected for seven days with puromycin and subsequently replated for determination of the proliferation rates. Results are presented as fold increase in cell number relative to time 0. B) PCR analysis of *Sp2cko/cko* MEFs containing a retroviral expression vector for Sp2 (*vSp2*). The primer combination P1, P2, P4, vSp2 was used for amplification of the *Sp2cko* allele (P1–P2, 790 bp), the *Sp2ko* allele (P2–P4, 550 bp) and the *Sp2* cDNA (P1-vSp2, 421 bp). C) Western blot analysis of Sp2 in control and Cre-infected *Sp2cko/cko* and *Sp2cko/cko-vSp2* MEFs. The asterisk indicates a cross-reacting band. To control for loading, the same blot was re-stained for Sp1.

To further substantiate the conclusion that Sp2 is essential for proliferation of MEFs, we performed rescue experiments (Figure 10). First we infected *Sp2cko/cko* MEFs with a retroviral expression vector for Sp2 (*pBABE-vSp2-puro*), using infection with the corresponding empty vector (*pBABE-puro*) as a control. The resulting *Sp2cko/cko-vSp2* MEFs expressed Sp2 ectopically in addition to endogenous Sp2. Cre-mediated ablation of endogenous Sp2 in *Sp2cko/cko*-*pBABE-puro* MEFs inhibited cell growth (compare -Cre and +Cre growth curves in Figure 10A). Retroviral Cre-infection of *Sp2cko/cko-vSp2* led to a complete depletion of the floxed *Sp2* alleles as well (Figure 10B). However, expression of ectopic Sp2 was unchanged (Figure 10C), and proliferation of these MEFs was affected much less severe (compare +Cre and +Cre+vSp2 growth curves in Figure 10A). These findings reinforce the conclusion that Sp2 plays an essential role in cell proliferation.

## Discussion

Sp2 was originally identified by virtue of the similarity of its zinc finger region with the DNA-binding domain of Sp1 [15]. It is expressed in many different human and mouse cell lines [16] suggesting wide-spread expression *in vivo*. Our Sp2 expression analysis in mouse embryos supports this notion. Mouse *Sp2* mRNA is detectable in embryonic stem cells and in all tissues during different stages of mouse embryogenesis, with exception of the heart. In Western blots, the mouse Sp2 protein appears as a double band. Whether this is due to posttranslational modifications or reflects splice variants is unknown at this stage.

Insertion of the *IRES-lacZ-Neo* cassette into the third intron of the *Sp2* gene resulted in aberrant expression of an *Sp2-lacZ-neo* fusion RNA in *Sp2lzn/lzn* mice. Although no Sp2 protein, neither full-length nor a C-terminal truncated fragment was detectable, it is very likely that the glutamine-rich N-terminal part of Sp2 is expressed at a very low level. This would explain why the *Sp2lzn/lzn* embryos display a markedly less severe phenotype when compared to *Sp2null* embryos. *Sp2lzn/lzn* embryos alive are found beyond E12.5 whereas disruption of the *Sp2* gene in *Sp2null* embryos causes severe developmental retardation and embryonic lethality before E9.5. The different phenotypes of *Sp2lzn/lzn* and *Sp2null* mice indicates that the glutamine-rich N-terminal part of Sp2 has a function independent of the C-terminal zinc finger domain that is believed to be responsible for DNA-binding [16].

No particular cell lineage or developmental process appears to be affected in *Sp2null* embryos. Rather, Sp2-deficiency causes a general cellular defect that precludes normal progression of development resulting in early embryonic lethality. This conclusion is consistent with the observation that MEFs derived from E9.5 *Sp2null* embryos did not grow in culture. Moreover, *Sp2lzn/lzn* MEFs derived from E13.5 embryos stopped growing in culture and repeatedly died after the first passage whereas MEFs from wild type and *Sp2wt/lzn* littermates grew normally. Finally, Cre-mediated ablation of Sp2 in *Sp2cko/cko* MEFs severely impaired proliferation that could be rescued by ectopic expression of exogenous Sp2. Altogether, these findings strongly suggest that Sp2 is essential for the autonomous growth of MEFs in culture. Further analyses will be necessary to unravel the cellular processes that are responsible for the growth arrest of Sp2-depleted MEFs and to identify key target genes of Sp2. Furthermore, the mice carrying floxed *Sp2* alleles described here will allow for the analysis of the role of Sp2 in distinct cell types and in adult animals.

In conclusion, we have shown that Sp2 is required for normal progression of embryonic development, and that it is essential for survival of mouse embryos after E9.5. Sp2-deficient MEFs fail to grow in vitro, strongly indicating that the lack of Sp2 causes a cell-autonomous proliferation defect in these cells. Our data further strengthen the notion that, despite their structural similarities, Sp1, Sp2, Sp3 and Sp4 have non-redundant important functions during mouse development. Future research in our laboratories will be aimed at understanding the molecular basis for these distinct functions. Conditional knockout alleles, such as described here for the *Sp2* gene, are essential tools to perform these studies.

## Materials and Methods

### Ethics Statement

Research involving mice have been conducted according to the German Animal Protection Law (Tierschutzgesetz). The application for the experiments was reviewed and approved by the responsible local authorities (Regierungspräsidium Giessen, reference number V 54 - 19 c 20/15 cMR20/27).

### Whole-Mount β-Gal Staining and *In Situ* Hybridization

Whole-mount β-galactosidase staining of E13.5 embryos was performed according to Tewari et al. [25]. Whole-mount RNA *in situ* hybridization was performed essentially as described [26] using BM purple (Roche) as color substrate. For *Sp2* mRNA detection, we used a 903 bp digoxygenin-labeled fragment of exon 3 (nucleotides 183 to 1085 of mouse *Sp2* cDNA) cloned in *pcDNA3*. Appropriate plasmids to generate probes for *Brachyury* (T) and *Nkx2-5* were obtained from Martin Eilers and Thomas Braun, respectively. The *GATA4* probe is described in [9].

### Generation of Sp2-Specific Antibodies and Immunoblot

A polyclonal antiserum against mouse Sp2 was generated by immunization of New Zealand White rabbits with recombinant full-length mouse Sp2 protein expressed in *E. coli* according to standard procedures. The antiserum was subsequently affinity-purified using immobilized recombinant Sp2. For immunoblots, whole cell extracts from MEFs were separated through 6% SDS-polyacrylamide gels and blotted to PVDF membranes. Primary antibodies were visualized with the Amersham ECL kit.

### Generation of *Sp2lzn* Mutant Mice

A cosmid clone containing exons 2 and 3 as well as flanking intron sequences of the murine *Sp2* gene was isolated from a mouse 129/ola library at the German Science Centre for Genome Research (RZPD). A targeting vector was designed to flank exon 3 of the *Sp2* gene and the selection marker with *loxP* sites as follows. As starting vector, we used the pPNT plasmid containing the *pgk*-driven neomycin resistance gene (*pgk-neo*) and the herpes simplex thymidine kinase (*hsv-tk*) gene [27]. *LoxP* sites flanking the *pgk-neo* cassette were inserted as synthetic *XbaI*-*SalI*-*LoxP*-*KpnI* and *NotI*-*XhoI*-*LoxP*-*SalI*-[*XhoI*] oligonucleotides. A 1.8 kb fragment of intron 3 obtained by PCR was cloned into the *EcoRI* site located between *pgk-neo* and *hsv-tk*. A fragment containing exon 3 and flanking intron sequences was cloned as a 1.6 kb *NotI*-*XhoI* PCR fragment upstream of the floxed *pgk-neo* cassette. A third *loxP* site was then inserted as *NotI*-*BamHI*-*LoxP*-[*NotI*] oligonucleotide. Subsequently, a 3.2 kb *Sp2* genomic fragment containing exon 2 and flanking intron sequences was inserted as *NotI*-*BamHI* PCR fragment. Finally, we replaced *pgk-neo* by an *SA-IRES-lacZ-neo-SVpA* cassette obtained as a 7.4 kb *SalI* fragment from pGT1.8Iresβgeo [28]. More detailed information concerning the generation of the targeting vector will be provided upon request. We verified all of the constructs by restriction analysis and sequencing. The functionality of the *loxP* sites in the final targeting vector was analyzed by appropriate restriction digests after transformation into the Cre-expressing *E. coli* strain 294-Cre [29].

The targeting construct was introduced into C57BL/6×129/Ola F1 hybrid ES cells by electroporation and G418 resistant clones were screened for homologous recombination by Southern blotting using a 387 bp fragment overlapping intron 3 and exon 4 (Figure 2A). ES cells with a targeted *Sp2* allele were injected into blastocysts. Blastocysts were transferred to pseudopregnant females and chimeric offspring were identified by PCR. Chimeric mice were crossed to heterozygosity resulting in *Sp2wt/lzn* mice.

### Generation of *Sp2null* Mutant Mice

To generate an *Sp2null* allele, heterozygous *Sp2wt/lzn* mice were mated with *CAG-Cre* mice [18]. The resulting heterozygous *Sp2wt/ko* mice were intercrossed to generate *Sp2null* mice.

### Generation of Conditional *Sp2cko/cko* Mice

Mosaic, early embryonic, ubiquitous Cre mice (*MeuCre40*) [22] were crossed with *Sp2wt/lzn* mice to remove the *LacZ-Neo* selection cassette from the Sp2 tri-*loxP*-allele. The F1 generation was screened for the presence of the *Cre* gene and partial recombination of the tri-*loxP* allele. Mice with partial and mosaic patterns, eg positive for *Cre* and the floxed exon 3 (primers P6–P7), were then mated with C57Bl/6 mice for segregation of the Cre-recombined alleles from *MeuCre40* in the F2 generation.

### Genotyping Embryos and MEFs by PCR

The following primers and amplicons were used for genotyping (see Figures 2, 5, 7 and 8 for orientation):

Primers:

P1: 5′-CCCTCTCAGAACTTTCAGATC-3′

P2: 5′-CTTAGGAGGGATCTAGACTAG-3′

P3: 5′- CATCGCCTTCTATCGCCTTCTTGA-3′

P4: 5′- ACCGAGAGCAAGTTCATGTC-3′

P5: 5′- GCTATTGCTCTTGTCTTTAGC-3′

P6: 5′-TATCCCTGCGGATCCATAACT-3′

P7: 5′-GGATACTTGCATTTGATCGGC-3′

vSp2: 5′-TCAGCCCACTGATAGTCAGG-3′

Cre 3′: 5′-CGATGCAACGAGTGATGAGGTTC-3′

Cre 5′: 5′-GCACGTTCACCGGCATCAAC-3′

Pex4: 5′-ATTCAGCTGCCATTCTCCGA-3′

Pex5: 5′-AGCCCACTGATAGTCAGGTT-3′

Amplicons:

*Sp2* wild type allele: P1–P2: 760 bp; P1–P5: 457 bp

*Lzn* allele: P2–P3: 950 bp

*Sp2null* allele: P2–P4: 550 bp

*Sp2cko* allele: P1–P5: 487 bp

*Lzn* and *Sp2cko alleles*: P6–P7: 620 bp

*pBABE-vSp2-puro*: P1-vSp2: 421 bp

*Sp2 cDNA*: Pex4-Pex5: 229 bp

### Northern Blot Analysis

Northern blot analysis was performed according to standard protocols with 20 µg of total RNA extracted from E18.5 embryos using a ^32^P-labeled 1.4 kb *Sp2* cDNA probe encompassing exons 2 to 5 (Figure 3B).

### Plasmids and Retroviral Infections

The *pBABE-Cre-puro* plasmid was previously described [30]. The retroviral *pWLZ-Cre-neo* plasmid was generated by cloning the *Cre* recombinase from *pBABE-Cre-puro* as a *[XhoI]-EcoRI* fragment into the *[BamHI]-EcoRI*-restricted *pWLZ* vector. The retroviral expression plasmid for Sp2 (*pBABE-Sp2-puro*) was obtained by cloning the full-length *Sp2* cDNA into the *BamHI* site of *pBABE-puro*.

High-titre viruses were produced by transient transfection of retroviral constructs into the Phoenix-Eco packaging cell line using FuGENE HD transfection reagent (Roche) according to standard procedures. Retrovirally infected MEFs were selected with appropriate antibiotics using the following concentrations: 2 µg/mL of puromycin and 1 mg/mL of G418.

### Determination of Growth Curves

MEFs were isolated from E9.5 and E13.5 embryos using standard methods. MEFs were immortalized by serial passages of primary MEFs. After retroviral infection MEFs were selected with appropriate antibiotics for seven days. Subsequently, 2.5×10^4^ cells were plated on 6-cm dishes and duplicates were counted after 2, 4 and 6 days.

### Histology

Embryos were dissected and a tail snip or the yolk sac was removed for genotyping. Embryos were fixed in Carnoy's solution (60% ethanol, 30% chloroform, 10% acetic acid) at 4°C overnight and embedded in paraffin according to standard procedures. Sections (8 µm) were stained with hematoxylin and eosin.
